# Investigation of Surface Integrity in Ultra-Precision Grinding of TiC-Reinforced Ti_3_SiC_2_ (MAX Phase Composite)

**DOI:** 10.3390/ma19132699

**Published:** 2026-06-23

**Authors:** Dennis Patrick Wilhelm, Anh Tuan Vu, Cornelia Rojacher, Thomas E. Weirich, Thomas Bergs

**Affiliations:** 1 Manufacturing Technology Institute (MTI), RWTH Aachen University, 52074 Aachen, Germany; t.bergs@mti.rwth-aachen.de; 2 Fraunhofer Institute for Production Technology IPT, 52074 Aachen, Germany; anh.tuan.vu@ipt.fraunhofer.de (A.T.V.); cornelia.rojacher@ipt.fraunhofer.de (C.R.); 3 Central Facility for Electron Microscopy (GFE), RWTH Aachen University, 52074 Aachen, Germany; weirich@gfe.rwth-aachen.de

**Keywords:** ultra-precision, grinding, MAX phase, surface integrity, mold, precision glass molding

## Abstract

Precision glass molding is an economical and resource-efficient method for manufacturing precision optics in a replicative way, offering advantages over conventional manufacturing methods, particularly for complex geometries. However, challenges arise due to different thermal expansion coefficients between the mold and the glass, which lead to shape deviations during the cooling process and require high compensation efforts. This study investigates the machining behavior during ultra-precision grinding of an innovative MAX phase composite whose coefficient of thermal expansion can be specifically adapted to that of glass. The aim is to evaluate the influences of varying process parameters and material configurations on surface integrity and the suitability of ultra-precision grinding for mold manufacturing in the context of precision glass molding. Systematic grinding tests were carried out and complemented by force measurements. The resulting surfaces were characterized using optical measurement technology and atomic force microscopy; in addition, the edge zone was analyzed using transmission electron microscopy. The results confirm the basic suitability of ultra-precision grinding for the MAX phase composite but point to potential subsurface damage that could limit its usability in precision glass molding.

## 1. Introduction

Modern markets such as data communication, medical technology, and optical sensing are driving an increasing demand for complex precision glass optics [1,2]. These optics are conventionally manufactured by grinding and polishing [3]. This machining process is particularly resource- and cost-intensive in the field of complex optical geometries [4]. Precision glass molding (PGM) is an alternative manufacturing approach in which a glass blank is positioned inside a process chamber between two molds that closely replicate the negative geometry of the optical element [5]. The process chamber is subsequently heated to a glass-specific molding temperature. At this specific temperature, it is possible to shape the glass without damage by bringing the mold halves together and transferring the geometry of the molds to the glass. The molding process is followed by controlled cooling. After that, the finished precision optics can be removed [6].

This manufacturing approach offers economic and ecological advantages over conventional machining, primarily due to its high scalability. A wafer-level approach enables the simultaneous production of multiple optics in a single process. This approach requires approximately the same amount of energy as molding a single optic (single cavity), significantly improving energy efficiency per component [7].

A particular challenge in PGM is meeting the stringent geometric specifications (surface integrity) required for precision optics and thus the molds at PGM. Typical guideline values are shape deviations on the order of PV-Error < λ/10 and surface roughness Sq < λ/100 for target wavelengths λ < 1 µm. Similarly, the machined molds must have a damage-free subsurface avoiding crack formation (under load, or even without) [8]. For applications in the visible wavelength range (λ ≈ 380–780 nm), this corresponds to shape deviations with PV-Error < 1 µm and surface roughness with Sq < 10 nm.

These specifications are challenged by process-related shape deviations caused by thermal expansion. During cooling, the glass, which generally has a higher coefficient of thermal expansion (CTE) than the mold material, undergoes greater shrinkage. Additional development is required to compensate for these shape deviations. This deviation is commonly compensated using finite element method (FEM) simulations to adjust the mold design, or through empirical correction loops during mold manufacturing [6,7].

This development effort reduces the cost efficiency of PGM and becomes particularly significant for non-rotationally symmetrical mold geometries, such as those used in wafer-level molds [9]. In such cases, complex and cost-intensive three-dimensional (3D) FEM simulations are required, demanding specialized expertise [10]. The efficiency of the PGM process chain could be significantly improved by using molds with a CTE tailored to the glass being molded.

Based on the current state of the art, binderless nanocrystalline tungsten carbide (n-WC) is predominantly used as a material for molds in PGM. Properties of the material, such as grain size (<100 nm) and crystalline composition (no binder, single-phase) are of particular importance [11]. Sub-micrometer-sized crystallites lead to tolerable defects in process-induced microstructural damage due to trans- or intracrystalline fractures. Similarly, the Hall–Peach effect increases grain boundary strength, which reduces stress-induced crack propagation [12]. Furthermore, a polycrystalline single-phase microstructure offers operating conditions that result in constant material removal and constant tool-to-material interaction. This has a significant influence on the achievable dimensional accuracy and roughness. These positive properties are offset by the CTE of α_200–500 °C_ ~ 4.5 × 10^−6^ K^−1^ of n-WC, which is low compared to the CTE of low-T_g_ glass (α_200–500 °C_ ≈ 7 × 10^−6^ K^−1^).

**Figure 1:**
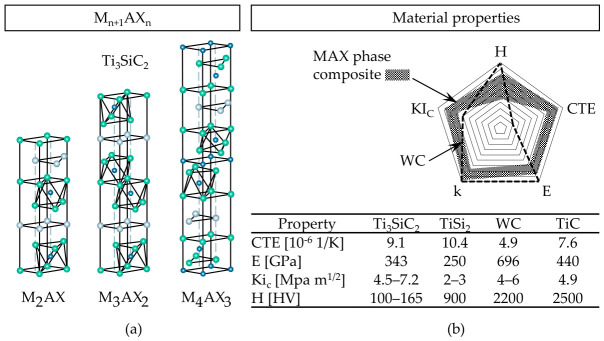
(**a**) Atomic structure of Ti_3_SiC_2_; (**b**) comparison of essential mechanical properties of Ti_3_SiC_2_ [13], TiSi_2_ [14], WC [15], and TiC [16]. High thermal conductivity (k) is relevant for PGM processes.

To achieve a CTE matching that of low-T_g_ glasses a MAX phase composite was developed. The material consists of a reinforcement phase TiC with a low CTE (α_20–500 °C_ ≈ 7.6 × 10^−6^ K^−1^) and second phase Ti_3_SiC_2_ from the MAX phase group with a high CTE (α_20–1000 °C_ ≈ 9.1 × 10^−6^ K^−1^). The term MAX phase is derived from the chemical composition M_n+1_AX_n_. M represents a transition metal from groups 3 to 6 in the periodic table, A represents an element from groups 13 to 16, and X is carbon or nitrogen. Ti_3_SiC_2_ was selected because it limits interdiffusion with the glass and the reinforcement phase. The unit cell of Ti_3_SiC_2_ exhibits an alternation of TiC octahedra and Si planes, resulting in a unique combination of typical ceramic and metallic properties [13]. Figure 1 shows the atomic structure of Ti_3_SiC_2_ as well as a comparison of selected material properties.

**Figure 2:**
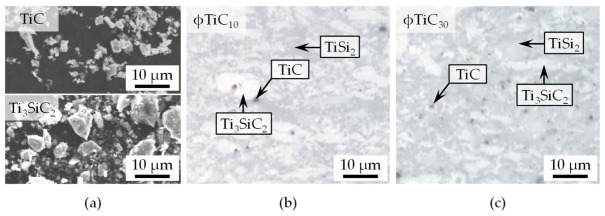
(**a**) SEM image of Ti_3_SiC_2_ and TiC powder particles; (**b**,**c**) light microscope images of Ti_3_SiC_2_ composites with TiC volume fractions of 10 vol.% (ϕTiC10) and 30 vol.% (ϕTiC30) [17].

The material was produced through field-assisted sintering, during which a third phase, TiSi_2_, forms. As a result, the final microstructure consists of Ti_3_SiC_2_, TiC, and TiSi_2_ phases distributed throughout the composite. Electron backscatter diffraction was used to determine the average grain sizes of the individual phases (0.41 µm for Ti_3_SiC_2_, 0.26 µm for TiC, and 0.59 µm for TiSi_2_). Representative microstructures are shown in Figure 2.

**Figure 3:**
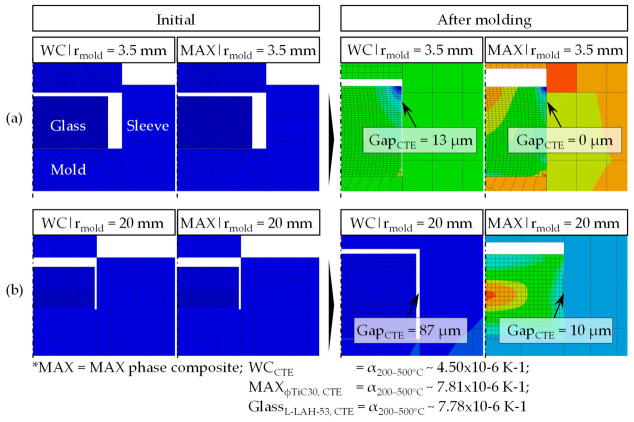
(**a**) Plain mold with a radius of 3.5 mm, glass (OHARA Corporation, type: L-LAH-53) before and after molding. (**b**) Plain mold with a radius of 20 mm, glass before and after molding.

The CTE of Ti_3_SiC_2_ is approximately twice that of n-WC. By reinforcing Ti_3_SiC_2_ with 10–30 vol.% TiC (ϕTiC), a composite CTE in the range of α_200–500 °C_ ≈ 8.2–7.8 × 10^−6^ K^−1^, can be achieved based on the linear mixture rule. Figure 3 illustrates the results of an FEM simulation that compares the dimensional deviations of two glass parts molded using different molds (r = 3.5 mm and r = 20 mm), taking into account the mold materials n-WC and MAX phase composite.

However, for common use in PGM applications, the machinability and achievable surface integrity of such MAX phase composites under ultra-precision (UP) grinding remain insufficiently understood. Therefore, this study investigates the influence of process parameters and material composition on the surface integrity.

## 2. Materials and Methods

All experiments were performed on a Moore Nanotech (Charlotte, NC, USA) 350FG Ultra-Precision Freeform Generator under temperature-controlled and vibration-isolated environment specifically for UP machining. The ambient temperature is maintained at ±0.1 K with a constant humidity of 50 ± 5%.

Grinding tools from the manufacturer Diamond Tools Mfg. Co., Ltd. (Tokyo, Japan). were used for UP grinding of the MAX phase composite. Previous studies demonstrated the suitability of these tools for machining this material [17]. Based on these findings, the tool configuration shown in Table 1 was selected.

The influence of process parameters on the surface integrity during UP grinding was investigated under defined kinematic conditions representative of rotationally symmetric mold manufacturing in PGM. The sample, mounted on the *c*-axis, rotates at a defined speed (n_w_), while the grinding tool moves along the *x*-axis (*y*-axis = 0) at a constant feed rate (v_f_) and constant rotational speed (n_c_), resulting in a spiral tool path. The relative cutting speed (v_c,rel_) is calculated in parallel with the equation:

v_c,rel_(D_w_, D_t_, n_w_, n_c_) = v_c_(D_t_, n_c_)-v_w_(D_w_, n_w_) (1)

where v_c,rel_ depends on the tool path diameter (D_w_) while the grinding tool diameter (D_t_ = 8 mm) remains constant. To maintain constant kinematic operating conditions, the rotational speed was adjusted according to the feed rate to maintain a constant path spacing of 1.6 µm. Under the most unfavorable conditions, the resulting deviation in cutting speed is approximately Δv_c_ ≈ 4%, which is considered negligible.

**Figure 4:**
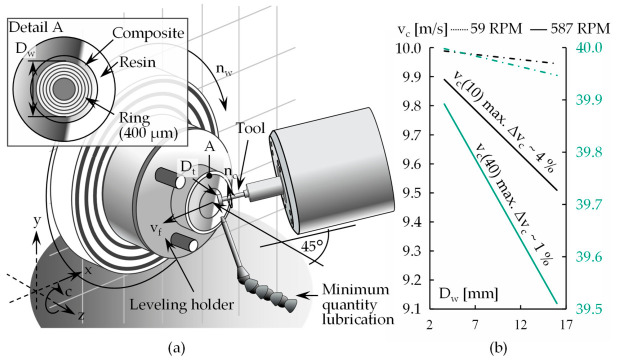
(**a**) Schematic representation of experimental setup and process kinematics; (**b**) resulting cutting speeds.

The samples were embedded in synthetic resin, polished flat (PV-Error < 1 µm, Sq < 15 nm) and aligned parallel to the x- and y-axes using a leveling holder and confocal displacement sensor (Keyence GmbH (Frankfurt am Main, Germany), type: CL-PT010). Ring-shaped grinding fields with a width of 0.4 mm were produced. For each sample, six rings were machined within a radial range of r = 3.6–8 mm. During machining, an isoparaffin-based lubricant was applied as a fine mist using a minimal quantity lubrication approach. To ensure consistent operating conditions and thus the comparability of the tests, the tool was re-dressed before each machining operation (applies to all experiments). Each experiment was repeated three times. Figure 4 represents the experimental setup and a graphical evaluation of the resulting cutting speeds.

Based on previous findings from other studies a full factorial experimental design (24 combinations) was used to investigate surface integrity [17]. Table 2 represents the varied factors and their values.

A laser confocal microscope (LCM, NanoFocus AG (Oberhausen, Germany), type: µsurf) was used to measure the 3D topographies, operating with a maximum magnification of 100x (measuring field: 160 µm × 110 µm). Furthermore, as this resolution is not sufficient to resolve individual grinding marks, selected areas were additionally analyzed using an atomic force microscope (AFM, Bruker (Billerica, MA, USA), type Dimension Icon). The parameter combinations for the AFM measurements are listed in Table 3. Two additional parameter sets (fields b and c) were investigated beyond the full factorial design, while fields a and d were selected from the experimental matrix.

The data from both measuring devices were processed and analyzed using the software Gwyddion (Version 2.62).

Structural damage to the mold is a critical factor for the use in PGM. The combined load of process forces (up to F = 40 kN) and temperatures (up to T = 800 K) can lead to material failure, particularly if the structure has been transformed. Breakouts from the mold might be one sign of this material failure [18]. To investigate the influence of machining on the microstructure in the area close to the surface (subsurface damage SSD), three focused ion beam (FIB) lamellae were taken from selected areas of a sample with a reinforcement phase of ϕTiC = 30 vol.%. These lamellae were analyzed using transmission electron microscopy (TEM) and complemented by energy-dispersive X-ray spectroscopy (EDX). One lamella was extracted from a polished, unground area and served as a reference, where minimal or no SSD is expected. The other two lamellae were taken from ground regions machined at v_c_ = 40 m/s. These represent two different load conditions: a low-load condition (a_p_ = 0.3 µm and v_f_ = 0.1 mm/min) corresponding to minimal chip thickness, and a high-load condition (a_p_ = 1 µm and v_f_ = 1 mm/min) representing the upper bound of the process-induced load.

In addition, grinding forces were measured to establish causal relationships with the observed surface integrity [19] (p. 79). A dynamometer (Kistler (Winterthur, Switzerland), type: MiniDyn 9256C1) was employed and mounted on a leveling holder, with the sample fixed on the measurement plate. Due to the cable connection of the dynamometer, rotation of the *c*-axis was not possible during these measurements. However, as the maximum deviation in cutting speed in the previous experiments was approximately Δv_c_ ≈ 4% in the most unfavorable case, the influence of this kinematics constraint is neglected. The machining path in this test setup follows a linear track (l = 0.5 mm) along the *x*-axis. Prior to machining, the sample was aligned parallel to the x- and y-axes according to the procedure described above. Grinding tools as given in Table 1 were used in this investigation. Each experiment was repeated three times.

The experimental design follows a one-factor-at-a-time method, in which individual parameters are varied while all others are kept constant. Table 4 presents the investigated parameter ranges.

## 3. Results

### 3.1. Grinding Tests

**Figure 5:**
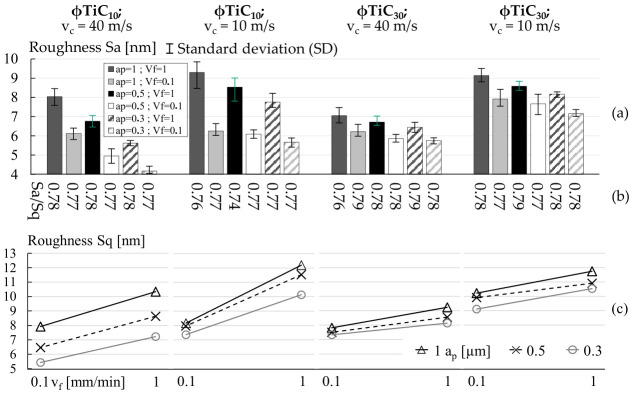
(**a**) Evaluation of surface roughness Sa in bar charts with standard deviation; (**b**) representation of ratio Sa/Sq; (**c**) representation of quadratic surface roughness Sq connected by 2D lines.

The measurement data generated by the LCM was processed using Gwyddion. The captured measurement fields were thus cropped to a size of 100 µm × 100 µm. To reduce surface waviness, a 13th-order polynomial filter was used. Following the surface roughness Sa and quadratic surface roughness Sq were calculated. The surface roughness parameters Sa and Sq, as well as their ratio (Sa/Sq), are summarized in Figure 5.

The measured values show that Sa ranges from 4.3 nm to 9.2 nm, while Sq varies between 5.5 nm and 12.2 nm. The ratio Sa/Sq remains within a narrow range of 0.74 to 0.79 across all experiments, indicating a consistent surface topography. The variation in roughness values reflects the influence of the applied process parameters.

The characteristic value Sq represents the square mean of the profile heights, while the characteristic value Sa represents the arithmetic mean of the profile heights. While Sa represents the average height deviation, Sq is more sensitive to pronounced peaks and valleys due to its quadratic definition. For surfaces with a normally distributed height profile—typically associated with ductile machining—the ratio Sa/Sq follows a characteristic value of [20]:

(2) $$\mathrm{Sa}\;=\;\sqrt{\left(\frac{2}{\pi }\right)}\;\cdot \;\mathrm{Sq}\;\approx \;0.8\;\cdot \;\mathrm{Sq}.$$

In contrast, brittle machining leads to non-normal height distributions due to fracture-induced surface features, resulting in deviations from this ratio.

**Figure 6:**
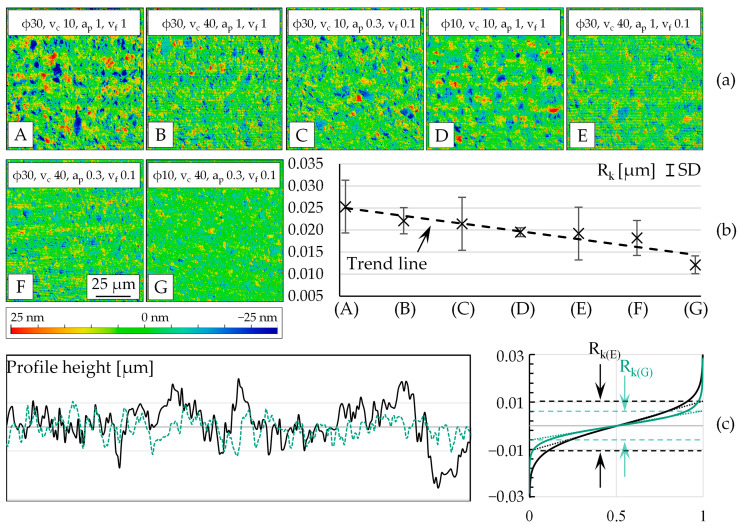
(**a**) False-color representation of selected topographies (height range: −25 nm to 25 nm); (**b**) comparison of core roughness R_k_; (**c**) example of two topography profiles (E and G) with associated Abbott curve and determination of R_k_.

In addition, selected surface topographies were analyzed by calculating the core roughness R_k_ using the Abbott curve [21]. Figure 6 shows representative measurement fields in a results-oriented order, indicating that height profile varying in a range from 25 nm to −25 nm (false-color).

The Abbott curve describes the distribution of profile heights as a function of material ratio and enables quantitative characterization of surface topography. Here, the material content is specified as a percentage in the definition range. The value range represents the profile height in micrometers. Figure 6c shows a comparison of two profile sections from measurements Figure 6a (E,G) as well as the corresponding Abbott curves. By applying a tangent at the center of the curves, the core roughness R_K_ can be read by extending them.

The chart in Figure 6b compares the evaluated topographies regarding the core roughness values. The analyzed data points show a range from R_k_ = 0.012 to R_k_ = 0.025 µm. The trend line indicates a downward trend in core roughness values over the course of the measurements.

A qualitative analysis of the measured topographies across Figure 6a (A–G) shows the trend in surface roughness in the false-color representation. The distribution of profile heights is visible by the color shift. Thus, the most pronounced color shift can be seen in Figure 6a (A). As core roughness decreases, this distortion of the profile height distribution systematically decreases until it reaches its minimum in Figure 6a (G). Thus, the determined core roughness correlates with the profile height distribution and, consequently, with the intensity of the color shift.

### 3.2. Grinding Normal Force

**Figure 7:**
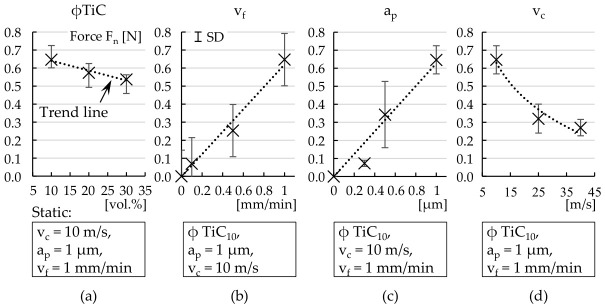
(**a**) Normal grinding force as a function of the reinforcement phase ϕTiC; (**b**) normal grinding force as a function of feed rate v_f_; (**c**) normal cutting force as a function of cutting depth a_p_; (**d**) normal cutting force as a function of cutting velocity v_c_.

The measured normal grinding forces as a function of the investigated process parameters are summarized in Figure 7.

The results show a clear dependence of the grinding force on both material composition and process parameters. Increasing the TiC reinforcement fraction from 10 to 30 vol.% leads to a reduction in normal force from F_n_ ≈ 0.65 N to 0.54 N. In contrast, increasing feed rate and cutting depth significantly increases the grinding force. The force rises from approximately F_n_ = 0.07 N at v_f_ = 0.1 mm/min to F_n_ = 0.65 N at v_f_ = 1 mm/min and similarly from F_n_ = 0.07 N at a_p_ = 0.3 µm to F_n_ = 0.65 N at a_p_ = 1 µm. Increasing cutting speed results in a reduction in grinding force, decreasing from approximately F_n_ = 0.65 N at v_c_ = 10 m/s to F_n_ = 0.27 N at v_c_ = 40 m/s.

### 3.3. Atomic Force Microscopy Measurments

**Figure 8:**
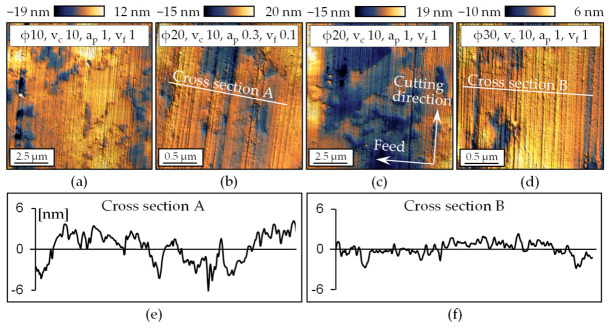
(**a**–**d**) Selected AFM topographies for different material compositions and process parameters; (**e**,**f**) corresponding cross-sectional profiles.

Figure 8 illustrates selected measurements and analyses from the AFM measurements.

The AFM images (Figure 8a–d) reveal surface topographies characteristic of ductile machining, with uniformly distributed grinding grooves and no evidence of brittle fracture. Variations in surface height corresponding to different microstructural phases are visible in the false-color representation. High-resolution images enable detailed analysis of the grinding grooves, whose average depths are found to be in the low single-digit nanometer range based on profile evaluations. The distribution of the profile heights, and thus the depth of the grinding grooves, is illustrated in Figure 8e,f using cross-sectional views.

### 3.4. Transmission Electron Microscopy Measurments

**Figure 9:**
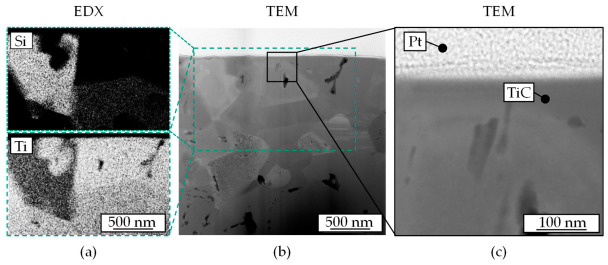
Analyses of initially polished surface, including (**a**) EDX elemental distribution of Ti and Si for phase differentiation; (**b**) TEM image of near-surface region; (**c**) Enlarged TEM image of the near-surface region.

Figure 9 presents the TEM analyses of the initially polished surface of the MAX phase composite before UP grinding. The TEM image shown in Figure 9b clearly reveals crystallites beneath the platinum protection layer that was deposited prior to FIB milling. To identify the elemental composition of individual crystallites, EDX analyses of the TEM image regions were performed additionally. Thus, Figure 9a presents the distribution of silicon (Si) and titanium (Ti). Based on the process of elimination, TiC crystallites (containing no Si), which constitute the reinforcement phase, can be identified. All other phases contain Si; an exact differentiation between these phases is not possible. Figure 9a shows a magnified section of Figure 9c containing only TiC. Figure 9b,c reveal a damage-free edge zone independent of the varying microstructure.

**Figure 10:**
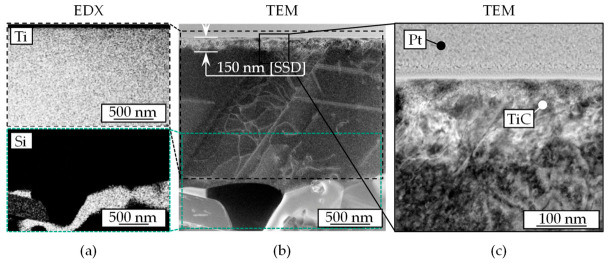
Analyses of ground surface (high chip thickness), including (**a**) EDX elemental distribution of Ti and Si; (**b**) TEM image indicating crack formation; (**c**) enlarged TEM image of the near-surface region.

The TEM analyses of a ground surface under conditions corresponding to high chip thickness are shown in Figure 10. The EDX results (Figure 10a) indicates that a TiC crystallite forms the boundary layer to the sample surface due to the absence of Siin the upper part of the image. In Figure 10b, bright transcrystalline cracks can be observed within the TiC crystallite, extending partially toward grain boundaries and terminating at adjacent phase interfaces. In addition, a modified subsurface zone is visible within a depth of approximately 100–150 nm beneath the surface. The TEM images (Figure 10c) reveal that the extent of SSD varies with depth, indicating a heterogeneous deformation behavior.

**Figure 11:**
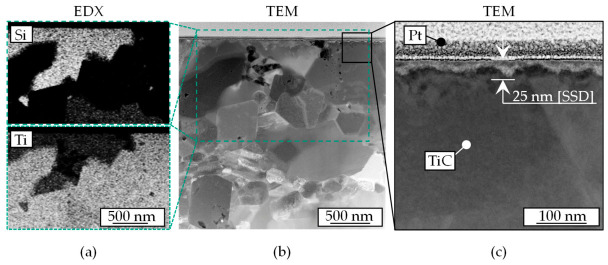
TEM analysis of a surface ground under low chip thickness, including (**a**) EDX elemental distribution of Ti and Si; (**b**) TEM image of the near-surface region; (**c**) enlarged TEM image of the near-surface region.

Figure 11 presents the TEM analysis of a surface ground under conditions corresponding to a theoretically low chip thickness (from the grinding experiments). The EDX results indicate the presence of different microstructural phases in the uppermost edge zone (Figure 11a). The edge zone highlighted in Figure 11c exhibits that SSD is observed primarily within TiC crystallites, where it extends to a depth of approximately 25 nm below the surface. In contrast, other microstructural components do not exhibit noticeable SSD in the analyzed region.

## 4. Discussion

The observed dependence of surface roughness on process parameters can be explained by their influence on the undeformed chip thickness. Increasing cutting speed reduces chip thickness due to shorter grain–workpiece contact time, resulting in finer grinding marks and lower roughness. In contrast, increasing feed rate and cutting depth leads to higher chip thickness, as the abrasive grains penetrate deeper and interact over longer distances with the surface, producing more pronounced grooves and increased roughness. These relationships confirm that the material removal rate, governed by the combination of process parameters, directly influences the resulting surface quality [22]. The results obtained in this study are consistent with established grinding theory, indicating that the machining behavior of the investigated MAX phase composite follows similar fundamental mechanisms [23,24,25,26]. Furthermore, the influence of the reinforcement phase fraction on the resulting roughness parameters was analyzed. The results indicate that an increase in the reinforcement phase fraction leads to an increase in the roughness parameters. A qualitative analysis of three-dimensional topographies provides a possible explanation for this behavior. Specifically, a comparison of the measurements in Figure 6a (A,D) highlights the effect of the reinforcement phase content on the profile height distribution. The corresponding color distribution shows that the profile height distribution is significantly more pronounced in measurement Figure 6a (A). Distinct color contrasts between individual regions suggest that certain structural components protrude above the mean surface level (z ≈ 0), while others are recessed below it. One possible explanation for this phenomenon is the variation in reinforcement phase fraction. Due to the so-called spring-back effect, the TiC crystallites—which are harder than the Ti_3_SiC_2_ matrix—are pressed into the softer binder during machining. Once the grinding tool passes over these regions and contact is lost, the TiC crystallites elastically recover and protrude from the binder matrix [27]. Consequently, a higher fraction of the reinforcement phase leads to a greater number of such protruding features, resulting in an increased variation in profile height distribution, and higher roughness values. Another explanation includes the differences in material removal rates between the individual microstructural components. Due to its higher hardness, TiC exhibits greater resistance to material removal compared to Ti_3_SiC_2_ and TiSi_2_, leading to locally elevated regions in the topography. Conversely, areas with increased material removal—indicated by lower regions in the height profile—may correspond to the softer Ti_3_SiC_2_ or TiSi_2_ phase. Furthermore, the influence of crystallographic orientation cannot be excluded. TiC, with its cubic crystal structure, is nearly isotropic and therefore only weakly dependent on machining direction. In contrast, TiSi_2_ (orthorhombic) and Ti_3_SiC_2_ (layered hexagonal) exhibit pronounced anisotropy, which may lead to orientation-dependent material removal behavior and contribute to the observed variations in surface topography.

The AFM images are used to study individual grinding marks in different microstructural components and their transition zones in detail. Although a direct spatial assignment of the individual phases (TiC, Ti_3_SiC_2_, and TiSi_2_) is not possible using AFM, it can be reasonably assumed that all phases are present within the analyzed 10 µm × 10 µm measurement fields based on the previously determined average grain size. The analysis shows that the characteristics of the grinding grooves are largely independent of the underlying microstructure. Only minor variations in groove morphology are observed across the measurement fields, and all grinding grooves exhibit feature characteristics of ductile material removal mechanism. This indicates that the presence and proportion of individual microstructural components do not significantly influence the chip formation under the investigated conditions. It can therefore be assumed that the critical chip thickness within the experimental limits is sufficiently low to ensure ductile machining behavior is achieved for all phases within the experimental parameter range [26]. Nevertheless, distinct topographical steps in the profile height are frequently observed at the grain boundaries between two phases. These steps are clearly visible in the false-color representations as abrupt changes in height. The profile analyses, shown in Figure 8e,f, exhibit that the grinding grooves themselves have depths of only a few nanometers (~1–4 nm), whereas height differences at phase boundaries can reach up to ~8 nm, and in some cases even ~20 nm (measurements not shown). These findings indicate that the overall surface topography is dominated less by the grinding grooves themselves and more by the microstructural transitions between phases.

The investigation of normal grinding forces provides further insight into the relationships between process parameters and the resulting surface topography. In particular, a clear correlation between the grinding force and core roughness is observed. Increasing the feed rate shows a monotonic increase in normal grinding force (higher material removal rate) [28,29]. The increase in normal grinding forces with feed rate is accompanied by a corresponding increase in core roughness. A comparable trend is also observed when varying the depth of cut is examined. Increasing the depth of cut also results in higher grinding forces due to the increased material removal rate and chip thickness [19] (p. 204) [30]. Accordingly, a correlation between cutting depth, grinding force, and core roughness is observed. In contrast, the influence of cutting speed on the grinding force is not monotonic [31]. The force decreases significantly between v_c_ = 10 m/s and v_c_ = 25 m/s, while only a slight reduction is observed at higher cutting speeds of v_c_ = 25 m/s and v_c_ = 40 m/s. This behavior resembles an asymptotic trend (v_f_ → ∞). One approach to explaining this phenomenon is based on the composition of the cutting force according to Lichun et al. [32]. Above a certain cutting speed, the proportion of frictional force begins to dominate over the proportion of cutting force. The chip thickness decreases to a point where its influence on the force system becomes negligible compared to the frictional force. In this range, the frictional force predominates, and its magnitude remains largely independent of the cutting speed. In addition to process parameters, increasing reinforcement phase fraction leads to a reduction in grinding force. This behavior can be explained by the heterogeneous microstructure of the material, which consists of a relatively soft binder matrix and a hard reinforcement phase. Under normal loading, the TiC crystallites are pressed into the softer surrounding matrix, effectively reducing the resistance encountered by the grinding tool. Consequently, the overall grinding force decreases with increasing reinforcement phase content.

TEM analyses are used to evaluate the near-surface region of the edge zone. This examination allows for the identification of process-induced changes in the material as compared to its initial state. These structural changes in the near-surface region can have a significant impact on the service life of molds in PGM. Under cyclic thermomechanical loading, pre-existing defects introduced during manufacturing, such as microcracks, may propagate progressively and eventually lead to material spallation or local fracture of the mold surface. The images of the sample with the polished surface (Figure 9), which depicts the initial state, do not reveal any visible SSD caused by the manufacturing process. The magnified image (Figure 9c) of the region containing the reinforcement phase serves as a reference for the following images, since in the MAX phase composite, the reinforcement phase exhibits the highest brittleness (H/K_IC_) and thus has the highest tendency toward brittle fracture [33]. A clearly defined affected zone can be seen in Figure 10b (grounded area). This zone extends ~150 nm into the material from the sample surface. This study cannot provide a clear identification of the damage within this zone. Nevertheless, a fragmentation zone (~150 nm) with induced microcracks may offer a possible explanation. In this process, the grinding process induces nanocracks, which lead to localized grain breakdown. Another approach that explains the origin of the damaged zone is based on the phenomenon of atomic structural transformation. The TEM images (Figure 10c) reveal amorphous atomic arrangements with isolated crystalline atomic chains remaining. A similar amorphization had already been observed by YIN et al. [34] during the UP grinding of a technical polycrystalline ceramic. In this study, the structural transformation is attributed to specific grinding forces and local thermal effects. The transformation within a crystallite, including dislocations, layer defects, and lattice distortions, was documented. The superposition of these effects results in complete lattice destruction. Below the marked SSD area in Figure 10b that ends sharply, the TiC crystallite is traversed by isolated transcrystalline cracks that terminate at the grain boundaries. This observation can be explained by the following hypothesis: The stresses induced in the material by the manufacturing process are not relaxed in the upper damaged zone. Relaxation occurs only below this zone within the crystallite. Here, relaxation is accompanied by crack formation in the shape of cleavage fractures, which is in some cases strongly direction dependent. This direction dependence follows energetically favorable planes. Since the grain boundaries themselves are not primarily damaged, crack propagation stops at the grain boundary. A comparison of Figure 10b and Figure 11b illustrates the influence of the grinding process on the extent of damage in the near-surface region. Within the boundaries of the experiment, it can be concluded that UP grinding of the MAX phase composite results in a visible effect on the near-surface region. It can also be documented that the degree of damage in the edge zone depends on the choice of process parameters. Here, the intensity of the damage depends on theoretical chip thickness. Greater chip thicknesses lead to more pronounced damage in the edge zone.

For the other microstructural components, Ti_3_SiC_2_ and TiSi_2_, no damage to the edge zone can be documented. This is evident in additional TEM images, which are not shown in this publication. Since the configuration of the MAX phase composite depends on the vol.% content of the reinforcement phase, a higher proportion of the reinforcement phase TiC results in a proportional increase in the damaged surface area of the edge zone.

## 5. Conclusions

The investigations conducted in this publication are intended to contribute to a general understanding of the machineability—through the manufacturing process of UP grinding—of the specific MAX phase composite. In particular, the focus of this work is on the influence of the reinforcing phase on machining, as this significantly defines the material’s technical suitability for use as a mold tool material for PGM. The investigations demonstrate the general suitability of UP grinding, yielding the following findings:

- UP grinding achieves surface roughness values down to Sq < 6 nm and Sa < 5 nm within the experimental limits.
- The proportion of the reinforcement phase TiC (vol.%) has a direct influence on the resulting surface roughness. As the TiC content increases, the roughness increases.
- If the process parameters are selected to result in a minimum theoretical chip thickness, this also leads to minimum roughness.
- The resulting roughness is not dominated by grinding scratches, rather by the profile height distribution of individual crystallites.
- The profile height distribution of the crystallites is directly dependent on the grinding normal force, which in itself is directly dependent on the process parameters and the proportion of the reinforcement phase. As the grinding normal force increases, the profile height distribution of the crystallites increases, and thus the achievable surface roughness.
- Within the limits of the experiment, it was not possible to achieve a damage-free edge zone using UP grinding. The damage affects exclusively TiC crystallites. Consequently, the proportion of the damaged surface increases with an increasing proportion of the reinforcement phase.
- The extent of the damage is directly dependent on the process parameters and thus also on the normal grinding force.

Based on the findings of this study, the MAX phase composite can be utilized (within the experimental limits) for an application in PGM in terms of its machinability with regard to the roughness parameters that can be achieved. While increasing the TiC content is beneficial for tailoring the CTE of the composite to match the requirements of PGM, the results indicate a tendency toward increased surface roughness. However, the results of this study show that these values remain within a range that is acceptable for use in PGM. Nevertheless, the proportion of the reinforcement phase correlates as well with the distribution of structural changes in the near-surface region. Therefore, further investigations are required to evaluate the long-term durability of the material and to determine to what extent these subsurface structural changes affect the service life of the molds used in PGM.

If the structural changes in the material caused by UP grinding are found to reduce mold lifetime in PGM, post-processing strategies such as corrective polishing may be investigated to remove the affected near-surface layer. In addition, future studies may explore optimized grinding conditions with reduced undeformed chip thickness in order to minimize subsurface damage during manufacturing.

## Figures and Tables

**Table 1:** Grinding tool configuration.

Abrasive Material	Average Grain Size	Bonding	Abrasive Concentration	Tool Shape
Diamond	#7000	MB1	C40	V-Shape
Systhetic	2.14 µm	(metal/resin)	(10 vol.%)	D = 30 mm

**Table 2:** Process parameters used in the full factorial experimental design.

Feed Rate v_f_ [mm/min]	Cutting Depth a_p_ [µm]	Cutting Velocity v_c_ [m/s]	Reinforcement Phase ϕTiC [vol.%]
0.1, 1	0.3, 0.5, 1	10, 40	10, 30

**Table 3:** Parameter combinations selected for AFM analysis.

Field	v_f_ [mm/min]	a_p_ [µm]	v_c_ [m/s]	ϕTiC [vol.%]
a	1	1	40	10
b	0.1	0.3	40	20
c	1	1	40	20
d	1	1	40	30

**Table 4:** Parameter ranges used for the grinding force measurements (one-factor-at-a-time design).

v_f_ [mm/min]	a_p_ [µm]	v_c_ [m/s]	ϕTiC [vol.-%]
Varied			
0.1, 0.5, 1	0.3, 0.5, 1	10, 25, 40	10, 20, 30
Constant			
ϕTiC = 10 vol.-%,	ϕTiC = 10 vol.-%,	ϕTiC = 10 vol.-%,	v_c_ = 10 m/s,
v_c_ = 10 m/s,	v_c_ = 10 m/s,	v_f_ = 1 mm/min,	v_f_ = 1 mm/min,
a_p_ = 1 µm	v_f_ = 1 mm/min	a_p_ = 1 µm	a_p_ = 1 µm

## Data Availability

The original contributions presented in the study are included in the article, further inquiries can be directed to the corresponding author.

## Abbreviations

The following abbreviations are used in this manuscript:

3D	Three-dimensional
AFM	Atomic force microscopy
CTE	Coefficient of thermal expansion
EDX	Energy dispersive X-ray spectroscopy
FEM	Finite element method
FIB	Focused ion beam
LCM	Laser confocal microscopy
n-WC	Nano grain tungsten carbide
PGM	Precision glass molding
SEM	Scanning electron microscope
SSD	Subsurface damage
TEM	Transmission electron microscopy
UP	Ultra-precision
